# An Integrated Approach to the Exposome

**DOI:** 10.1289/ehp.1104719

**Published:** 2012-03-01

**Authors:** Martie van Tongeren, John W. Cherrie

**Affiliations:** Institute of Occupational Medicine Edinburgh, United Kingdom, E-mail: martie.vantongeren@iom-world.org

The editorial by Lioy and Rappaport (2011) provides a timely addition to the discussion about the exposome and exposure science. We are encouraged by the recognition of the importance of combining measurements of personal exposure with measurements of biological markers of exposure. However, rather than focusing on two approaches (i.e., top-down vs. bottom-up), we advocate a fully integrated approach to measurement of the exposome.

There are currently serious limitations in measuring internal and external exposure. It may be feasible to measure biological markers in blood or other media periodically, but such measures are not without difficulties. Recent developments in omics technology are very promising, but many of these techniques have low reproducibility between laboratories, show high intraindividual variability, and are still expensive; in addition, uncertainties remain in biological interpretation of these markers (Vineis et al. 2009). It is still often impractical to prospectively measure personal inhalation, dermal, and ingestion exposure. Such information could be collected periodically, but the scientific effort would be great and the intrusion into the subjects’ lives would probably be unacceptable. Increased research effort will undoubtedly help improve measurement of both internal and external exposure. However, other sources of information exist that could contribute to constructing the exposome.

We all routinely leave traces of our exposome in everyday electronic databases or databases that could be easily constructed. For example, the goods we purchase in a supermarket are often tracked by loyalty cards, which may provide a rich source of information on food consumption and the consumer products we use. Consumption of electricity and natural gas is increasingly being logged electronically by utility companies to assist billing. These data could be used to determine use of electrical items (informative about exposure to electric and magnetic fields) and activity patterns. It is relatively straightforward to track movements of individuals using mobile phones, and these data can be used, for example, to help estimate exposure to air pollutants.

Within the next few years we will see an explosion in availability of sensors for many environmental contaminants that will be relatively cheap and easy to use and that could provide a more or less continuous log of information that can be related to exposure. These include simple sensors in the homes of subjects that continuously record information on air temperature, airborne contaminants, and other environmental factors. These sensors may provide personal exposure data or could, in combination with activity patterns and behavior, be used to reconstruct exposure profiles.

The availability of data on use and activity patterns, as well as developments in sensor and omics technology, suggests that the dichotomy in top-down and bottom-up approaches may not be appropriate, as there are other strategies that could be used to determine the exposome. In addition, the terms “top-down” and “bottom-up” may be interpreted differently, with consequent confusion of terminology. Instead, we should aim to develop a concept of the exposome that takes into account all sources of available exposure information.

The key factor in developing an integrated approach will be the articulation of clear theoretical paradigms linking exposures with disease. All of the exposure and contextual data could be used to reconstruct the exposome of individuals in an epidemiological study using appropriate models to link the data to parameters of interest in the exposome. Data on internal and external exposures, data on personal behavior, and environmental information from sensors could be used to triangulate on the exposome.

This is an extremely exciting time in exposure science, and we believe that the coming years will provide a great opportunity to make a significant leap forward in understanding the relationship between environmental exposure and disease. Maximizing the opportunities provided by various developments requires a fully integrated approach to the exposome. This approach must be based on a clear theoretical framework that incorporates measurement and modeling of external exposure, databanks on patterns of behaviors, and markers of internal exposure.
